# Menopausal hormone therapy: a systematic review of cost-effectiveness evaluations

**DOI:** 10.1186/s12913-017-2227-y

**Published:** 2017-05-05

**Authors:** Louiza S. Velentzis, Usha Salagame, Karen Canfell

**Affiliations:** 1 Cancer Research Division, Cancer Council New South Wales, Sydney, NSW Australia; 2 Breast and Gynaecological Cancers, Cancer Australia, Surry Hills, Sydney, NSW Australia; 3 School of Public Health, Sydney Medical School, University of Sydney, Sydney, NSW Australia; 4 Prince of Wales Clinical School, The University of New South Wales, Sydney, NSW Australia

**Keywords:** Cost-effectiveness, Systematic review, Menopausal hormone therapy, Breast cancer

## Abstract

**Background:**

Several evaluations of the cost-effectiveness (CE) of menopausal hormone therapy (MHT) have been reported. The aim of this study was to systematically a﻿nd critically review economic evaluations of MHT since 2002, after the Women’s Health Initiative (WHI) trial results on MHT were published.

**Methods:**

The inclusion criteria for the review were: CE analyses of MHT versus no treatment, published from 2002-2016, in healthy women, which included both symptom relief outcomes and a range of longer term health outcomes (breast cancer, coronary heart disease, stroke, fractures and colorectal cancer). Included economic models had outcomes expressed in cost per quality-adjusted life year or cost per life year saved. MEDLINE, EMBASE, Evidence-Based Medicine Reviews databases and the Cost-Effectiveness Analysis Registry were searched. CE evaluations were assessed in regard to (i) reporting standards using the CHEERS checklist and Drummond checklist; (ii) data sources for the utility of MHT with respect to menopausal symptom relief; (iii) cost derivation; (iv) outcomes considered in the models; and (v) the comprehensiveness of the models with respect to factors related to MHT use that impact long term outcomes, using breast cancer as an example outcome.

**Results:**

Five studies satisfying the inclusion criteria﻿ were identified which modelled cohorts of women aged 50 and older who used combination or estrogen-only MHT for 5-15 years. For women 50-60 years of age, all evaluations found MHT to be cost-effective and below the willingness-to-pay threshold of the country for which the analysis was conducted. However, 3 analyses based the quality of life (QOL) benefit for symptom relief on one small primary study. Examination of costing methods identified a need for further clarity in the methodology used to aggregate costs from sources. Using breast cancer as an example outcome, risks as measured in the WHI were used in the majority of evaluations. Apart from the type and duration of MHT use, other effect modifiers ﻿for breast cancer outcomes (for example body mass index) ﻿were not considered.

**Conclusions:**

This systematic review identified issues which could impact the outcome of MHT CE analyses and the generalisability of their results. The estimated CE of MHT is driven largely by estimates of QOL improvements associated with symptom relief but data sources on these utility weights are limited. Future analyses should carefully consider data sources and the evidence on the long term risks of MHT use in terms of chronic disease. Th﻿is review ﻿highlights the considerable ﻿difficulties in conducting cost-effectiveness analyses in situations where short term benefits of an intervention must be evaluated in the context of long term health outcomes.

## Background

Menopausal hormone therapy (MHT) is considered an effective treatment for menopausal symptoms [1]. However, to assess whether MHT is cost-effective in any group, benefits for quality of life need to be considered together with the health risks, and resources (costs) associated with use. Conceptually, the cost-effectiveness (CE) of any intervention can be summarised using a single measure known as the cost per Quality-Adjusted Life Years (QALY). This measure can be estimated using modelled evaluation of the benefits, costs, and any adverse health effects (short or long term﻿) of the intervention. However, information on the CE of MHT is limited, in part because MHT was widely available for a number of years before standardised national health technology assessm﻿ent processes involving CE evaluation were established. In the 1980s and 1990s, a few CE analyses of MHT were performed [2–4], but these included assumptions (e.g. that MHT prevented cardiovascular disease) that have not been supported  by subsequent findings [5]. Partly as a consequence, the findings of these early CE evaluations were favourable [2–4]. As new evidence to better quantify the effects of MHT has emerged, updated estimates of the overall benefit and cost trade-offs for MHT continue to be of potential value since MHT is still relatively widely used in many developed countries. For example, in Australia, 13% of women in their fifties and sixties reported being current users in a national survey conducted in 2013, and of these 73% had been using MHT for 5 years or longer [6]. Ongoing evaluation of the CE of drugs that are widely used, ensures that optimal health investments continue to be made.

Evidence of the health risks associated with MHT use has been accumulating from epidemiological studies and trials in the late 1990s and early 2000s [7–15]. However, in 2002, the Women’s Health Initiative (WHI) estrogen plus progestin trial was stopped early after an increased risk of breast cancer, coronary heart disease (CHD), stroke, and pulmonary embolism was reported in study participants randomised to combination MHT compared to women randomised to placebo [16]. Since then, evidence of these health risks has been provided by additional studies including a large UK study [17, 18]. Although the interpretation of these findings has been disputed [19], independent reviews [5, 20] of the worldwide evidence from other trials and observational studies by regulatory authorities continue to support cautious targeted use. For example, the UK Medicines and Healthcare products Regulatory Agency (MHRA) concluded that ‘for all women the lowest effective dose should be used for the shortest possible time’ and ‘the need to continue MHT should be reviewed at least yearly taking into consideration the change in balance of risks and benefits’ [5]. A review of randomized controlled studies of MHT versus placebo by the US Preventive Services Task Force found the risks of taking MHT, when used to prevent chronic conditions, outweigh the benefits [21]. This resulted in the US Preventive Services issuing recommendation statements against the routine use of MHT for the prevention of chronic conditions in postmenopausal women, above 50 years of age [20, 22].﻿ In 2015 clinical guidelines on the menopause were commissioned by the National Institute for Heal﻿th and Care Excellence (NICE) to provide advice for healthcare professionals and women on the menopause and symptom relief [National Institute for Health and Care Excellence. Menopause: diagnosis and management of menopause. (NICE guideline 23.) 2015. www.nice.org.uk/guidance/ng23].﻿ NICE recommended the adoption of an individualised approach at all stages of diagnosis, investigation and management of menopause; taking this into acco﻿unt, one recommendation was to offer women MHT for vasomotor symptoms after discussing with them the short-term (up to 5 years) and longer-term benefits and risks. A﻿s part of the NICE guideline, a review of economic evaluations of short term treatments for menopausal symptoms and a de novo modelled evaluation were﻿ performed. ﻿﻿The NICE guidelines did not provide quantit﻿ative summary estimates of risks and benefits [Hickey M, Banks E. NICE guidelines on the menopause. BMJ. 2016;352:i191﻿], although these had been earlier provided by the MHRA for chronic disease outcomes [5].

The overall aim of the current study was to conduct a systematic review of evaluations on the CE of MHT and to harness standardised frameworks for reporting standards and key model parameters. We confined the review to 2002 and later since the WHI 2002 report marked the beginning of a substantial change in public and clinician understanding of the overall risks associated with MHT use, and led to large scale reductions in MHT use in many developed countries [23, 24]. Evaluations conducted in the 1980s and 1990s were not included in this review because some assumptions used in their models have not been supported by subsequent findings. Using the CHEERS checklist and the Drummond framework [25] each identified study was assessed in terms of data sources for MHT-related utility with respect to symptom relief; the methodology for assessing costs; outcomes considered in the economic models and the comprehensiveness of the models in respect to factors that affect particular chronic disease outcomes, using breast cancer risk as an example. With respect to breast cancer, we assessed whether evaluations considered the type of MHT, the duration of use, the impact of body mass index and the timing of initiation of MHT in relation to the menopause, since the evidence supports these factors as modifiers of the relative risk (RR) of breast cancer in relation to MHT use [11, 17, 26, 27].

## Methods

### Search strategy, eligibility criteria and article selection

A systematic search was conducted for relevant articles published from 2002, with the date of final search on 23^rd^ February 2016. Databases searched were Ovid MEDLINE (US National Library of Medicine, Bethesda, MD, USA), EMBASE (Reed Elsevier PLC, Amsterdam, The Netherlands), Cost-effectiveness Analysis Registry (Tufts Medical Centre, Boston, MA) and Evidence-Based Medicine Reviews (American College of Physicians, Wiley-Blackwell, New Jersey, US), which contains the NHS Economic Evaluation Database, the Health Economic Evaluations Database and other Cochrane Library databases. Search terms used (as specified for MEDLINE) were ‘hormone therapy’ , OR ‘hormone replacement therapy, OR (hormon$ or estrogen or oestrogen) adj (treatment or therp$)’ , OR ‘hormone substitution’ AND ‘cost’ , OR ‘cost-utility’ , OR ‘cost-effective$’ , OR ‘costs and cost analysis’ [explode]. Terms were searched in all fields. Searches were limited to those conducted in humans and in females, with no language or other restrictions. Reference lists of identified papers were also searched for further relevant source articles. The search strategy was based on Cochrane Review recommendations [28].

Inclusion criteria were a priori defined as: CE or cost-utility analyses of MHT verses no treatment, in a population of healthy women, considering a range of long term health outcomes related to MHT use (breast cancer, coronary heart disease, stroke, fractures, colorectal cancer [5]﻿﻿)﻿ and menopausal symptom relief, with outcomes expressed in cost per quality-adjusted life year (QALY) or cost per life year. Exclusion criteria were analyses conducted for women with a pre-existing condition or a higher risk for a disease than the general population, analyses without inclusion of MHT-related long-term health outcomes or exclusion of the beneficial effect of menopausal symptom relief, or articles estimating only net costs of MHT use. Two investigators (LV, US) independently conducted the searches, reviewed titles and abstracts followed by the full texts of selected publications according to eligibility criteria and extracted data from studies using a structured form. Disagreements and queries at each stage of this process were resolved by discussion with a third investigator (KC).

Eligible publications were assessed for the completeness of their reporting using the 24-item CHEERS (Consolidated Health Economic Evaluation Reporting Standards) checklist [29] developed by the International Society for Pharmacoeconomics (ISPOR). Full adherence to any item recommendation was noted as ‘yes’ , with partial adherence as ‘incomplete’ and non-adherence as ‘no’. Eligible papers were also assessed for methodological quality using a 36-point checklist [30] which is based on the Drummond checklist [25]. The checklist considers the elements of study design, data collection, analysis and result interpretation which are expected in a sound economic evaluation.

### Assessment of data sources for MHT-related utility

The quality of life benefits or ‘utility’ associated with menopausal symptom relief following MHT use is formally accounted for in a cost-effectiveness assessment via inclusion of utility scores. These scores allow the impact of the quality of life benefit to be quantitatively assessed. The source data for the utility values for menopausal relief used in each CE assessment were documented and assessed. If more than one publication was referenced by the CE analysis the methodology for combining utility values from different studies was considered.

### Assessment of costing methods used in primary studies

The evaluation of the cost-effectiveness of MHT depends on the quality of the source data for all relevant health care costs in both the ‘no treatment’ scenario, and in the MHT treatment scenario, including all downstream costs relating to MHT-related outcomes. To evaluate the methodology for assessing costs, each primary CE evaluation was examined for sources of unit costs, method of cost aggregation and country of study. If multiple references were cited for costs related to a particular health outcome, then the method of identifying costs (e.g. literature review or expert opinion) as well as the process used by authors to aggregate costs, was also extracted. In addition, the CE evaluations were examined in terms of presentation of separate unit costs and resource quantities and whether ranges of costs were provided to reflect varying degrees of disease severity or staging, where applicable. Any additional costing issues identified in each evaluation were also noted.

### Assessment of particular health outcomes included in economic models

Using the MHT-related health outcomes identified in the synthesis of the worldwide data on MHT risks and benefits conducted by the UK MHRA [5], the following chronic diseases were assessed for inclusion in the economic models: breast cancer, colorectal cancer, CHD, deep vein thrombosis, endometrial cancer, fractures, ovarian cancer, pulmonary embolism, stroke and venous thromboembolic disease. Other health outcomes associated with MHT for which some randomised control trial evidence exists [21] were also assessed in our review for completeness, but these did not contribute to the current assessment of model quality. These included: urinary incontinence [31]; gallbladder disease [32]; and dementia [33]. Data sources from which the relative risk was chosen for each outcome, were assessed for each included CE assessment.

### Assessment of the comprehensiveness of the models

To further evaluate the comprehensiveness of the CE evaluations with respect to the completeness of their representation of disease outcomes, breast cancer was used as an example outcome. Factors for which evidence supports a role as an effect modifier for breast cancer outcomes i.e.: duration of MHT use, MHT type, body mass index and time of initiating MHT in relation to menopause, were examined for inclusion in CE models. Relative risks for breast cancer associated with estrogen-only and estrogen plus progestin MHT were assessed and the source data documented.

### Ethics approval

This article is a systematic and critical review of economic evaluations of the cost-effectiveness of MHT and therefore Ethics Committee approval was not required.

## Results

### Selection of studies

**Fig. 1:**
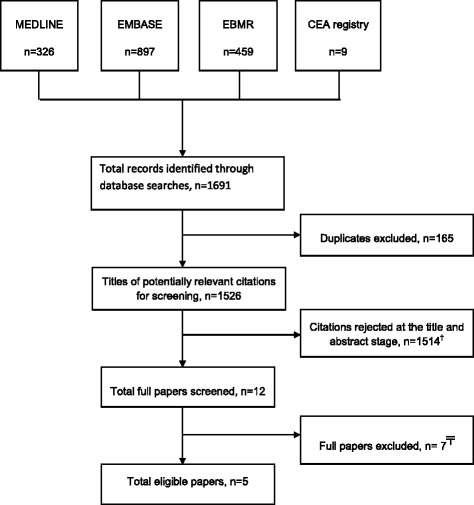
Study selection flow chart. ^†^Excluded articles referred to other hormone/endocrine therapies (e.g. growth hormone, fertility medication, adjuvantcancer therapy) for conditions unrelated to the menopause, osteoporosis related medication, non-hormonal interventions for menopause, costs related to health service utilisation by MHT users, review articles, opinion pieces and other articles unrelated to menopausal hormone therapy. ^╤^Excluded articles referred to osteoporosis or increase risk of fracture, excluded disease events, or did not report required health outcomes. EBMR: Evidence-Based Medicine Reviews. Additional files legend: Search strategies for CE of MHT

Figure 1 summarises the search process conducted. Electronic literature searches originally identified a total of 1691 citations. After accounting for duplicates, 1526 publications remained. Of these, 1514 were rejected when the title and the abstract were reviewed and found not to be relevant (e.g. referred to other hormone/endocrine therapies such as growth hormone, fertility medication or adjuvant cancer therapy). The full text of the remaining 12 publications was examined. Seven articles were excluded for the following reasons: not based on analysis for healthy women but relevant only to a subgroup of women with osteoporosis or at an increased risk of fracture [34–36]; not including a range of  MHT-related chronic disease outcomes in the economic model [37, 38], and estimation of net costs of MHT use rather than a CE analysis [39]. One article [40] compared MHT use versus no therapy, but was conducted from the perspective of osteoporosis prevention. The evaluation considered the annual costs and outcome impacts from the use of MHT, or raloxifene or alendronate (agents for primary prevention of osteoporosis), by postmenopausal women over a 7 year period. This article was excluded from the review because its economic model did not include the beneficial effect of menopausal symptom relief on the calculated QALYs and considered only three clinical outcomes: fractures, myocardial infarction and breast cancer. The article concluded that MHT use resulted in net harm, although this was not based on a full assessment of the harms and benefits of MHT use. The remaining five articles [41–45] met the inclusion criteria and were included in the systematic review. ﻿It﻿ should be noted that after our initial search was completed a CE evaluation of ﻿MHT and non-MHT interv﻿entions for alleviating vasomotor symptoms ﻿in menopausal women was undertaken for ﻿NICE and was published [National Institute for Health and Care Excellence. Menopause: Appendix L-Health Economics. (NICE guidelines 23). 2015. https://www.nice.org.uk/guidance/NG23/documents/menopause-appendix-l2]. However, this evaluation was not included in our review because the included outcomes (which were agreed with a Guidelines Development Group)﻿ included vasomoter symptoms, vaginal bleeding, d﻿iscontinuat﻿ion of treatment, breast cancer, and venous thromboembolism, but not other chronic disease outcomes. The explicit focus was ﻿o﻿n short-term MHT use (5 years or less) and on the comparison with other treatment alternatives﻿ for short term use and therefore, the scope was somewhat distinct from the included analyses in our review.

### Summary characteristics

Table 1 summarises some of the characteristics, main assumptions and key findings of the included MHT CE evaluations. Two evaluations were conducted in the US and three in Europe, each modelling cohorts of women using MHT at the age of 50 years and over, for durations of use of between 5 and 15 years. All included studies used the assumption that combination MHT or estrogen-only MHT were exclusively used by women with or without a uterus, respectively. Four models considered a time horizon of 50 years or over the lifetime of the cohorts, whereas one evaluation considered a period of 9 years. All evaluations used a 3% discount rate and willingness to pay thresholds which were in line with country-specific guidelines. All studies had at least one author who declared a potential for perceive﻿d conflict of interest (i.e. were employed by or had accepted speaker or consulting fees, or funding for the evaluation or writing assistance from an organisation which could reasonably be seen to have an interest in the findings of the evaluation).

**Table 1:** Summary table of study characteristics

Author year	Country	Perspective	Time horizon	MHT duration base-case	MHT type	Discount Rate (%)	Year of costs	Cost per QALY	CE?	CIF?
Salpeter et al. 2009 [41]	USA	Societal	lifetime	15 years	E + P&E (pooled data)	3	2006	Age 50: $2,438;	Yes	Yes
								Age 65: $27,953	No	Yes
Lekander et al. 2009 [42]	UK	Health care	50 years	5 years	E + P(+U) E(-U)	3	2006	Age 50:		Yes
								£580(+U)^a^	Yes	
								£205(-U)^a^	Yes	
Lekander et al. 2009 [43]	USA	Societal	50 years	5 years	E + P(+U) E(-U)	3	2006	Age 50:		Yes
								$2,803(+U)	Yes	
								$295(- U)	Yes	
Ylikangas et al. 2007 [44]	Finland	Health care	9 years	9 years	E + P(+U)	3	2003/2004	Age 50–70:		Yes
								€2,996(+U)	Yes	
								(≤5 y MHT);		
								€4613(+U)	Yes	
								(≤9 y MHT);		
Zethraeus et a.l 2005 [45]	Sweden	Societal	50 years	5 years	E + P(+) E(-U)	3	2003	Age 50:		Yes
								SEK 12,807 (+U)	Yes	
								SEK 8,266(- U);	Yes	
								Age 55:		Yes
								SEK 10,844 (+U)	Yes	
								SEK 7,960(- U);	Yes	
								Age 60:		Yes
								SEK 9,159(+U)	Yes	
								SEK 11,043(-U);	Yes	

*Abbreviations: CE* cost-effective, *CIF* declared potential for perc﻿eived ﻿conflict of interest, *E* estrogen-only MHT, *MHT* menopausal hormone therapy, *P* progestin, *+ U* women with a uterus, *-U* women without a uterus

^a^Equivalent amounts in US dollars: $1072 (£580) and $379 (£205). Rate = 1.8485, 30/06/2006

Overall, the evaluations concluded that MHT use by women 50-60 years of age was below the indicative willingness-to-pay threshold of the country for which the analysis was conducted; thus all evaluations concluded that MHT could be cost-effective. Although the incremental cost-effectiveness ratio (ICER) values from each evaluation cannot be directly compared due to difference in currencies and the year for which costs were obtained, the three most recent evaluations [41–43], which all used 2006 costs, found that the cost per QALY for MHT use by 50 year old women ranged from $744 to $2,803 US dollars for non-hysterectomised women and from $263 to $295 US dollars for hysterectomised women, as at April 2017 (Table 1). These estimated ICERs are well under the relevant indicative willingness to pay threshold (which in US context, for example, is $50-100,000 US dollars). Even in older women (>65 years), one evaluation found that MHT would have an ICER of less than $30,000 US, which is again less than the relevant indicative willingness to pay threshold [41].

The assessment of the included evaluations according to the CHEERS checklist [29] is presented in Table 2. Although the historical context of the included primary studies must be borne in mind, incomplete reporting was observed for all included evaluations, and in many cases reporting was incomplete across most of the reporting domains in CHEERS (title/abstract, introduction, methods, results, discussion and other). Table 3 presents the findings of the quality assessment of the included evaluations using an extended Drummond checklist [30]. The included evaluations met most checklist criteria in the study design section, however, for all evaluations nearly half of the criteria for data collection, analysis and interpretation of results were either partially addressed or not addressed.

**Table 2:** Assessment of economic evaluations according to the CHEERS reporting guidelines

Item No	Section^a^	Salpeter et al. 2009 [41]	Lekander et al. 2009a [42]	Lekander et al. 2009b [43]	Ylinkangas et al. 2007 [44]	Zethraeus et al. 2005 [45]
	Title and abstract					
1	In title identify study as an economic evaluation, or use relevant terms & describe interventions compared	incomplete	incomplete	incomplete	incomplete	incomplete
2	Provide structured summary in the abstract with specified subheadings	incomplete	incomplete	incomplete	incomplete	incomplete
	Introduction					
3	Provide explicit statement of broader context, state study question & relevance for public policy or practice decisions	incomplete	incomplete	incomplete	incomplete	incomplete
	Methods					
4	Target population: describe base case population & subgroups analysed including why they were chosen	incomplete	incomplete	incomplete	yes	incomplete
5	Setting and location: state relevant aspects of the system(s) in which the decision(s) need(s) to be made	no	no	no	no	no
6	Describe the study perspective & relate this to the costs evaluated	yes	incomplete	yes	incomplete	incomplete
7	Describe the comparators and state why they were chosen	incomplete	incomplete	incomplete	yes	incomplete
8	State the time horizon(s) over which costs & consequences are evaluated & say why appropriate	incomplete	incomplete	incomplete	incomplete	incomplete
9	Report the choice of discount rate(s) used for costs & outcomes; say why appropriate	incomplete	incomplete	incomplete	incomplete	incomplete
10	Describe choice of health outcomes & their relevance for the type of analysis performed	incomplete	yes	yes	yes	incomplete
11	Describe fully the methods used for identification of included studies & synthesis of clinical effectiveness data	incomplete	incomplete	incomplete	incomplete	incomplete
12	Describe the population & methods used to elicit preferences for outcomes	incomplete	incomplete	incomplete	incomplete	incomplete
13	Describe approaches & data sources for estimating resource use; describe research methods for valuing resource items as unit cost	incomplete	incomplete	incomplete	incomplete	incomplete
14	Give dates of est resource quantities & unit costs, methods for adjusting unit costs to year of reported costs & details regarding conversion to common currency base & exchange rate	incomplete	incomplete	incomplete	no	incomplete
15	Describe choice of model & reason for use. Provide figure of model structure.	yes	incomplete	yes	not applicable	incomplete
16	Describe all structural or other assumptions underpinning the decision-analytical model	yes	yes	yes	yes	yes
17	Describe methods for dealing with data issues, pooling data, extrapolation, model validation/adjustments & uncertainty	Incomplete	incomplete	incomplete	incomplete	incomplete
	Results					
18	Report values, ranges, references & probability distributions for all parameters & reasons/sources for distributions used for uncertainty	yes	incomplete	incomplete	incomplete	incomplete
19	For each intervention report mean values for estimated costs & outcomes, mean comparator differences & if applicable ICERs	yes	incomplete	incomplete	incomplete	yes
20	Describe uncertainty effect for parameters & uncertainty in relation to model structure and assumptions	incomplete	incomplete	incomplete	incomplete	incomplete
21	Describe effect of heterogeneities on cost, outcome or CE	yes	yes	yes	incomplete	yes
	Discussions					
22	Summarise findings, how they support conclusions, fit with current knowledge & their generalisability. Describe limitations	incomplete	incomplete	incomplete	incomplete	incomplete
	Other					
23	Describe sources of funding, role of funder & any other non-monetary sources of support	Yes	incomplete	incomplete	incomplete	incomplete
24	Describe potential for conflict of interest as per journal policy or as per recommendations of International Committee of Med Journal Editors	yes	yes	yes	yes	yes

^a^Recommendations have been condensed to fit the table. A full description of the recommendation corresponding to each item number of the CHEERS checklist can be found at: https://www.ispor.org/workpaper/CHEERS/revised-CHEERS-Checklist-Oct13.pdf

**Table 3:** Quality assessment of economic evaluations according to the Drummond checklist

		Economic evaluation				
Quality assessment parameter		Salpeter et al. 2009 [41]	Lekander et al. 2009a [42]	Lekander et al. 2009b [43]	Ylinkangas et al. 2007 [44]	Zethraeus et al. 2005 [45]
Study Design	Was the research question stated?	Yes	Yes	Yes	Yes	Yes
	Was the economic importance of the research question stated?	No	Partially	Partially	Yes	Partially
	Was the viewpoint of the analysis clearly stated and justified?	Yes, but not justified	Yes, but not justified	Yes, but not justified	Not clearly stated, not justified	Yes, but not justified
	Was a rationale reported for the choice of the alternative programmes or interventions compared?	N/A	N/A	N/A	N/A	N/A
	Were the alternatives being compared clearly described?	Yes	Yes	Yes	Yes	Yes
	Was the form of economic evaluation stated?	Yes	Yes	Yes	No	Yes
	Was the choice of form of economic evaluation justified in relation to the questions addressed?	No	Yes	Yes	No	Yes
Data Collection	Were the sources of effectiveness estimates used stated?	Yes	Yes	Yes	Yes	Yes
	Were details of the design and results of the effectiveness study given?	Yes	Yes	Yes	Yes	Yes
	Were the primary outcome measures for the economic evaluation clearly stated?	Yes	Yes	Yes	Yes	Yes
	Were the methods used to value health states and other benefits stated?	No	Yes	Yes	Partially	No
	Were the details of the subjects from whom valuations were obtained given?	No	No	No	Partially (only age)	No
	Were productivity changes (if included) reported separately?	Not stated	N/A	Not stated	N/A	Not stated
	Was the relevance of productivity changes to the study question discussed?	No	N/A	No	N/A	No
	Were quantities of resources reported separately from their unit cost?	No	No	No	No	No
	Were the methods for the estimation of quantities and unit costs described?	No	No	No	No	No
	Were currency and price data recoded?	Yes (aggregate prices)	Yes (aggregate prices)	Yes (aggregate prices)	Yes (aggregate prices)	Currency yes; price data only for MHT
	Were details of price adjustments for inflation or currency conversion given?	Yes, for inflation. Prices from other countries used. No, for currency conversion	Yes, for inflation. No other currency used	Yes, for inflation. Prices from other countries used. No, for currency conversion	No	Yes, for inflation. No other currency used
	Were details of any model used given?	Yes	Yes	Yes	N/A	Yes
	Was there justification for the choice of model used and the key parameters on which it was based?	Yes for model. Partially for parameters	Partially for model & for parameters	Yes for model. Partially for parameters	N/A	Partially for the model. Yes for parameters
Analysis & Interpretation	Was time horizon of cost and benefits stated?	Yes	Yes	Yes	Yes	Yes
	Was the discount rate stated?	Yes	Yes	Yes	Yes	Yes
	Was the choice of rate justified?	No	No	No	No	No
	Was an explanation given if cost or benefits were not discounted?	Both were discounted	Both were discounted	Both were discounted	No, benefits not discounted	Not clear what was discounted
	Were the details of statistical tests and confidence intervals given for stochastic data?	Standard deviations given for QALYs	No	No	No	No
	Was the approach to sensitivity analysis described?	Yes	Yes	Yes	Yes	Yes
	Was the choice of variables for sensitivity analysis described?	Yes	Yes	Yes	Yes	listed only in results table
	Were the ranges over which the parameters were varied stated?	Yes	Yes	Yes	Yes	Yes
	Were relevant alternatives compared?	Yes	Yes	Yes	Yes	Yes
	Was an incremental analysis reported?	Yes	Yes	Yes	Yes	Yes
	Were major outcomes presented in a disaggregated as well as aggregated form?	Yes	No	No	No	Yes
	Was the answer to the study question given?	Yes	Yes	Yes	Yes	Yes
	Did conclusions follow from the data reported?	Yes	Yes	Yes	Yes	Yes
	Were conclusions accompanied by the appropriate caveats	Partially	Partially	Partially	Partially	Partially
	Were generalisability issues addressed?	Partially	Partially	Partially	Partially	Partially

*Abbreviation: N/A*: not applicable

### Data sources for MHT-related utility

Three studies [42, 43, 45] used MHT-related utility values from one study [46]. These CE evaluations used the QOL improvement of women with mild menopausal symptoms and the QOL improvement of women with severe menopausal symptoms to produce an average utility value to inform their models. In the Finnish CE evaluation [44] the MHT-related utility value was based on data from a randomized intervention trial of estrogen and progestin. The trial was conducted in Finland, where 419 women were randomised to six parallel treatment groups, receiving 4 dose combinations with no placebo group [47, 48]. The economic evaluation was conducted for three of the four dose combinations and therefore data were restricted to 279 study participants.﻿ The 15D health related QOL instrument was used at year 6 and 9 of the study by which time participants had dropped to 210 and 58 at years 6 and 9, respectively. The CE evaluation did not provide any details about which QOL utility values were used from the randomised trial, nor how results were aggregated from the various treatment groups. In the remaining CE evaluation [41] a willingness-to-pay survey and articles with different preference classification systems were used to produce a MHT-related utility score although the method used to generate this score was not detailed.

All studies assigned health state disutilities to chronic disease conditions associated with MHT use in their model accounting for the QOL decrements associated with potential adverse effects of MHT use [5]. However, ﻿in two studies [42, 43] assumptions were made on the disutilities of having colorectal cancer and VTE (0.9 assigned to both conditions) but these﻿ were not subsequently varied in sensitivity analyses.﻿ In another study [44], disutilities due to chronic diseases were not mentioned in the methods nor were they presented, although results of a sensitivity analysis varying the disutility score of all events was presented.

### Summary of costing methods and other costing issues

All evaluations used treatment costs reported from other studies. Two evaluations based treatment costs on studies conducted in the same country as the economic evaluation [42, 45] whereas the remaining articles derived costs from a combination of studies from the same country and others [41, 43, 44]. No evaluation provided complete information on how data sources were selected from the available literature, nor was the methodology used to combine the data from different sources described. No evaluations provided unit costs or resource quantities. In some evaluations [42–45] costs were referenced to previously conducted CE evaluations or other secondary studies instead of primary references, making it difficult to trace the original data sources. Presentation of a range of costs according to the seriousness or stage of a disease also varied between evaluations; one [41] provided both an average cost and ranges for all outcome categories, two studies had ranges for some diseases such as fracture and breast cancer [42, 43] whereas another [44] provided a single cost estimate for each disease outcome. Three evaluations [42, 43, 45] did not conduct any sensitivity analysis for MHT-associated costs (except varying the discount rate) and one evaluation [44] tested one higher and one lower value for costs associated with some chronic diseases (breast cancer, colon cancer and stroke) but only one evaluation conducted one-way sensitivity analysis and probabilistic sensitivity analysis [41]. Two evaluations were conducted from the perspective of health care [42, 44] considering direct costs and three from a societal prospective [41, 43, 45]. One evaluation [44] used direct medical costs associated with the first year of treatment for all chronic diseases evaluated and no long-term costs. One [42] assumed that there were no long-term direct costs associated with VTE and vertebral fractures. In another evaluation, [43] it was not clear how the authors selected direct costs for breast cancer treatment based on the single source article referenced; the source article estimated costs for women aged 65 years and older with early-stage breast cancer. Another article [45] did not present values for any treatment costs included in their model and therefore it was not possible to compare costs used in the evaluation against the source references provided. A total cost per age (50, 55 and 60 years old) was given for MHT-users and women not using MHT, according to whether they had a uterus or were hysterectomised, and regardless of age, the difference in health costs associated with women not using MHT versus MHT users were SEK 9,739–13,645 (as at April 2017 equivalent to ~ €1,014–1,420; ~US $1,100–1,541).

### MHT health related outcomes

MHT-associated health outcomes and data sources included in the CE evaluations are presented in Table 4. All evaluations considered the following as separate outcomes: breast cancer, colorectal cancer, CHD, stroke, pulmonary embolism, deep vein thrombosis and fractures, except one evaluation [41] where deep vein thrombosis was not considered, and CHD and stroke were considered as a compound outcome. None of the identified studies explicitly included ovarian cancer as an outcome. None of the evaluations incorporated other effects of MHT use, such as gallbladder disease. Three out of 5 evaluations [42, 43, 45] used WHI data to inform relative risks for the included health outcomes, one evaluation [41] used data from trials (including the WHI) and observational studies whereas one study used its own trial data [44].

**Table 4:** MHT-related health outcomes and data sources of relative risks included in reviewed evaluations

MHT-related health outcomes	*Publications*				
	*Salpeter et al. 2009* [41]	*Lekander et al. 2009a* [42]	*Lekander et al. 2009b* [43]	*Ylikangas et al. 2007* [44]	*Zethraeus et al. 2005* [45]
Breast Cancer	WHI (2002, 2004); meta-analysis of studies (2000, 2002); Cochrane database systematic review, 2005; HERS, 1998; MWS, 2003; Collabo-rative reanalysis of data on MHT and breast cancer, 1997; follow-up data from BCDDP, 2000; Danish cohort study, 2004; meta-analysis of WHI + HERS + WEST, 2002;	WHI (2003, 2006)	WHI (2002, 2004)	Events from intervention study: 279/ 419 women allocated to 3 of 4 dose combinations of EV&MA	WHI (2002, 2004)
Colorectal Cancer	WHI (2002, 2004); review of studies, 2002; meta-analysis of WHI & HERS 2002; CPS-II, 1995; pooled RR from HERS I, II&WHI, 2004; meta-analysis of studies, 1999	Two WHI study publications (2004)	WHI (2002, 2004)	Events from intervention study (see above)	WHI (2002, 2004)
Coronary Heart Disease (CHD)	Cardiovascular events presented rather than CHD: NHS (2001, 2002); WHI (2002,2003, 2004, 2006, 2007); meta-analyses (1991, 2000, 2002, 2004, 2005); case control study 1991-1994, HERS,1998;	WHI (2007)	WHI (2002, 2004)	Events from study (see above)	WHI (2002, 2004)
Deep vein thrombosis (DVT)	Not included	Included under VTD	Included under VTD	Events from study (see above)	Included under VTD
Dementia	Not included	Not included	Not included	Not included	Not included
Endometrial Cancer	Not applicable	Not applicable	Not applicable	Not specified but﻿ events from the study﻿ possibly﻿ included	Not applicable
Fractures (hip, vertebral and other osteoporotic)	WHI (2002,2006); Cochrane systematic review, 2005; Danish osteoporosis prevention study, 2000; literature review, 2001; New Zealand study assessing bone density and factors determining bone loss, 2002; Danish study on MHT effect on fractures, 2006;	WHI (2003, 2006)	WHI (2002, 2004, 2006)	Events from study (see above)	WHI (2002, 2004)
Gallbladder disease	Not included	Not included	Not included	Not included	Not included
Ovarian Cancer	Not included	Not included	Not included	Not specified but ﻿events from the study possibly included	Not included
Pulmonary Embolism (PE)	WHI (2002, 2004); two meta-analyses, 2002; data from a decision model of short term MHT on symptom relief, 2004;	Included under VTD	Included under VTD	Events from study (see above)	Included under VTD
Stroke	Stroke was considered together with CHD as one disease category	WHI (2007)	WHI (2002, 2004)	Events from study (see above)	WHI (2002, 2004)
Urinary incontinence	Not included	Not included	Not included	Not included	Not included
Venous thrombo-embolic disease (VTD)	PE events included but not DVT	WHI (2004, 2006)	WHI (2002, 2004)	Covered by DVT and PE	WHI (2002, 2004)

*Abbreviations: BCDDP* Breast Cancer Detection Demonstration Project, *CPS-II* Cancer Prevention Study II, *EV* estradiol valerate *HERS* The Heart and Estrogen/Progestin Replacement *Study, MA* medroxyprogesterone acetate, *MWS Million Women Study, NHS* Nurses Health Study, *WEST trial* Women's Estrogen for Stroke Trial, *WHI* Women’s Health Initiative

### Comprehensiveness of models

The relative risk of breast cancer associated with MHT used in three articles [42, 43, 45] varied according to the type of MHT modelled (oestrogen-only or combination). For one evaluation [41] breast cancer risks for both MHT types were pooled together. Three studies considered use of MHT for 5 years [42, 43, 45], however, of the two studies [41, 44] that considered MHT use for longer than 5 years, only one [41] increased the relative risk with increasing time periods of MHT use. Body mass index (BMI) and time of initiating MHT in relation to the menopause were not considered in any of the CE evaluations.

## Discussion

To our knowledge, this is the first time economic evaluations of the CE of MHT have been critically reviewed in a systematic manner, using a standardised framework. Five evaluations, identified since 2002, met the inclusion and exclusion criteria. Although all evaluations included consideration of breast cancer, colorectal cancer, stroke, CHD, and fractures in their outcomes, none included the full range of known MHT-associated health effects which have been summarised in independent regulatory reviews [5]. Assessment of the evaluations using the CHEERS and Drummond checklists identified incomplete reporting in various categories which hindered effective review and interpretation of study findings. For women 50 to 60 years of age, all evaluations found MHT to be cost-effective and below the indicative willingness-to-pay threshold of the country for which the analysis was conducted. ﻿Our findings must be interpreted in historical context wi﻿th respect to the included studies - firstly, reporting standards for economic evaluations have improved over time and since the publication of the primary studies; and secondly, quantitative independent syntheses of the long term risks﻿ and benefits of MHT were not necessarily a﻿vailable at the time of some of the primary study analyses (for example, th﻿e quantitative synthesis by the independent regulatory agency, the UK MHRA, was published in 2007). Nevertheless, our findings do influence the interpretation of the results of the included primary studie﻿s, since we identified reporting and methodological issues with the evaluations which could impact the outcome of MHT CE analyses and the generalisability of their results.

The current systematic review has certain limitations. It should be noted that only the main parameters of economic models were evaluated, rather than all parameters. For example, only factors related to breast cancer risk were reviewed when assessing the comprehensiveness of the models. However, breast cancer is one of the most important disease outcomes related to MHT use and effect modifiers of this association have been well documented. Issues similar to those identified from the current methodological evaluation for breast cancer may also apply to the other MHT-related health outcomes, although further investigation would be required to confirm this. The current review was also constrained by the limitations of the included studies. There was incomplete reporting for a number of parameters as identified by the quality assessment tools used, especially in terms of costing methodology and presentation of disaggregated costs, to enable a more detailed quality review. However, despite these limitations a number of findings have been identified that are constructive and informative for future CE evaluations.

In addition to choosing the appropriate health outcomes, factors that can modify the relationship between the intervention and a health outcome, need to be carefully considered in the model’s structure. We chose to assess the comprehensiveness of the CE evaluations for MHT by specifically examining the modelling of effect modification related to MHT-associated breast cancer risk. Although MHT type and varying duration of use were considered in some evaluations, body mass index and the timing of MHT initiation in relation to the menopause [17, 26, 27] were not accounted for. A body of evidence exists to suggest that the risk of breast cancer is greater in thinner MHT users than overweight or obese users (for example one large scale analysis found ﻿﻿for ﻿﻿estrogen-only MHT: ﻿RR 1.65 (95% CI 1.54-1.76) for BMI <25 kg/m^2^; RR 1.22 (95% CI 1.15-1.30) for BMI ≥25 kg/m^2^; combined MHT: RR 2.20 (95%CI 2.11-2.30) for BMI <25kg/m^2^; RR 1.81 (95%CI 1.73-1.9) for BMI >﻿25kg/m^2^) [27]. We suggest that future evaluations should consider all effect modifiers when modelling the CE of MHT use and in sensitivity analyses.

A favourable CE outcome for MHT is driven by its effects, or potential effects, on menopausal symptom relief, fractures, and potentially colorectal cancer [16]. Of these, the alleviation of menopausal symptoms could be considered the principal reason women would be using MHT and therefore the utility value (preference) related to MHT would be the main driver of QOL which would increase the cost-effectiveness of CE. Three evaluations [42, 43, 45] used utility values from a single study [46]. In addition to its small sample size (*n* = 104) and limited assessment (2 time-trade off questions asked), this study was conducted in Sweden and use of its data may not be widely applicable, as utility values have previously been shown to vary between countries [49–51]. Quality of life improvements for menopausal symptom relief will act to increase the CE o﻿f MHT use.  Conversely, the adverse health effects will act to decrease the cost-effectiveness. We suggest that future CE evaluations should consider all these effects in their models and carefully consider data sources for utility weights.

Given the significant costs associated with the treatment of chronic disease outcomes related to MHT use, it is important that costs are accounted for appropriately in a CE analysis of MHT. As treatment patterns, treatment availability and clinician preferences can differ [52] across health systems and countries [53] and methods used to collect costs can also vary (e.g. micro-costing, case-mix grouping, use of charges), aggregating the results of different economic evaluations requires a clear methodology which explains how overall findings were calculated. It is also essential that the unit costs for each resource, resource quantities and methods for aggregating costs from various sources are provided in any economic evaluation [25] for clarity and transparency. Details of costing methods also need to be provided so that costs included in models for CE analyses can be verified. In addition, the potential effects of MHT on cos﻿ts related to outcomes such as gallbladder disease, urinary incontinence and dementia were not included in the CE evaluations performed to date.

The ﻿literature search for the current systematic review was conducted at a similar time as that for the literature review of economic evaluations for the NICE clinical guidelines on menopause [National Institute for Health and Care Excellence. Menopause: diagnosis and management of menopause. (NICE guideline 23.) 2015. www.nice.org.uk/guidance/ng23]. ﻿The review by NICE aimed at finding economic evidence relating to short term treatments for menopausal symptoms, and included tibolone in addition to estrogen-only MHT and combination therapy. The studies that wer﻿e identified in the NICE review were assessed for their relevance to one of the clinical questions posed by an expert reference group (the Guidelines Development Group) which was ‘what is the most clinical and cost-effective treatment for the relief of individual menopause-related symptoms for women in menopause’. ﻿The conclusion of the review was that ‘no published health economic literature was identified that addressed the breadth of treatment alternatives included in the network meta-analysis for this guideline’. NICE then commissioned a de novo economic evaluation, which, as previously noted, had a differe﻿nt focus and different outcome criteria to those included in the current review, according to our pre-specified primary study inclusion criteria.﻿ This﻿ evaluation assessed the CE of 5 years of use of MHT, non-MHT drugs and other interventions for alleviating vasomotor symptoms in menopausal women a﻿ged 50 years old. It ﻿was concluded that ‘the model suggests that transdermal oestradiol and progestogen was the most cost-effective treatment in women with a uterus and that is reflected in the recommendation of this guideline. However, the Guidelines Development Group didn’t think the evidence was sufficiently strong to completely overturn clinical practice and the use of much cheaper oral oestadiol and progestogen as the principle first line treatment.’ The evaluation also concluded that ‘non-oral oestradiol was cost-effective in women without a uterus although this model relied more heavily on extrapolated data. The guideline recommendations for women without a uterus mirror the recommendations for women with a uterus with a choice given between the use of oral and transdermal preparations with the same rati﻿onale.’

As was the case for the NICE review, some of our identified primary studies focused on use of MHT for periods of not more than 5 years. In interpreting the findings at a population level, however, it should be borne in mind that substantial numbers of women continue to use MHT for durations of longer than 5 years. For example, in one recent study in Australia, three-quarters of current-users had used MHT for ≥5 years [6]. Therefore, deriving a picture of actual cost-effectiveness at a population-level requires consideration of the actual use of MHT in that population﻿.

## Conclusions

In conclusion, this critical assessment of cost-effectiveness evaluations of MHT identified a range of methodological issues affecting the interpretation of their findings and incomplete reporting of parameters which hindered effective review and transparency. ﻿Our findings must be interpreted in historical context with respect to the work presented in the included studies - firstly, reporting standards for economic evaluations have improved over time; and secondly, quantitative independent syntheses of the long term risk and benefits of MHT were not necessarily available at the time of some of ﻿the primary study analyses (for example, the quantitative synthesis by the independent regulatory agency, the UK MHRA, was published in 2007). Nevertheless, our findings do influence the interpretation of the results of the included primary studie﻿s. ﻿ Our﻿ review emphasises the considerable ﻿difficulties in conducting cost-effectiveness analyses in situations where short term benefits of an intervention must be evaluated in the context of long term health outcomes.﻿We recommend that any future cost-effectiveness assessments of MHT consider the current indications for use and the current recommendations by regulatory agencies for cautious targeted use; at the same time, a population-level assessment of cost-effectiveness should optimally account for the actual proportion of long-duration users (5 years or more), and account for the consequent impact on the risks of chronic disease in this group﻿. We also recommend that future evaluations consider the full range of known beneficial and harmful health outcomes and consider the established effect modifiers for such health outcomes. Comprehensive costing and health state utility studies should be performed to support future evaluations, and we recommend that these studies account for all health outcomes for which there is an established association with MHT use. In addition, the ISPOR CHEERS statement as elaborated in the task force guidance report should also be adhered to, so as to facilitate interpretation of findings and effective comparison of future cost-effectiveness assessments of MHT [29].

## Abbreviations

BMI: Body mass index
CE: Cost-effectiveness
CHD: Coronary heart disease
CHEERS: Consolidated health economic evaluation reporting standards
ICER: Incremental cost-effectiveness ratio
MHRA: Medicines and Healthcare products Regulatory Agency
MHT: Menopausal hormone therapy
NICE: National Institute for Health and Care Excellence
QALY: Quality-adjusted life year
QOL: Quality of life
RR: Relative risk
VTE: Venous thromboembolic event
WHI: Women’s Health Initiative.
